# Direct enhancement of viral neutralising antibody potency by the complement system: a largely forgotten phenomenon

**DOI:** 10.1007/s00018-023-05074-2

**Published:** 2024-01-11

**Authors:** Jack Mellors, Miles Carroll

**Affiliations:** Centre for Human Genetics and the Pandemic Sciences Institute, Nuffield Department of Medicine, University of Oxford, Oxford, UK

**Keywords:** Complement system, Neutralisation assays, Enhancement, Neutralising antibodies, Non-neutralising antibodies

## Abstract

Neutralisation assays are commonly used to assess vaccine-induced and naturally acquired immune responses; identify correlates of protection; and inform important decisions on the screening, development, and use of therapeutic antibodies. Neutralisation assays are useful tools that provide the gold standard for measuring the potency of neutralising antibodies, but they are not without limitations. Common methods such as the heat-inactivation of plasma samples prior to neutralisation assays, or the use of anticoagulants such as EDTA for blood collection, can inactivate the complement system. Even in non-heat-inactivated samples, the levels of complement activity can vary between samples. This can significantly impact the conclusions regarding neutralising antibody potency. Restoration of the complement system in these samples can be achieved using an exogenous source of plasma with preserved complement activity or with purified complement proteins. This can significantly enhance the neutralisation titres for some antibodies depending on characteristics such as antibody isotype and the epitope they bind, enable neutralisation with otherwise non-neutralising antibodies, and demonstrate a better relationship between in vitro and in vivo findings. In this review, we discuss the evidence for complement-mediated enhancement of antibody neutralisation against a range of viruses, explore the potential mechanisms which underpin this enhancement, highlight current gaps in the literature, and provide a brief summary of considerations for adopting this approach in future research applications.

## Introduction

Neutralisation assays remain the gold standard for measuring neutralising antibody potency. They are commonly used to determine key criteria for: investigating correlates of protection (CoP), evaluating vaccine-induced and naturally acquired immune responses, identifying new therapeutics, determining antibody specificity, and optimising existing therapeutic regimes [1–5]. The complement system—a humoral component of innate immunity—can directly impact neutralisation titres and the interpretations made from conventional neutralisation assays. Common practices such as the heat-inactivation of plasma samples, or the use of EDTA tubes for blood collection, inactivates the complement system. Furthermore, non-heat-inactivated samples can have varying levels of complement activity. The replenishment of complement activity with a characterized exogenous source of plasma, or with purified complement proteins, can greatly enhance antibody neutralisation with the potential to result in statistically significant correlations between in vitro and in vivo findings.

The complement system is a heat-labile component of the innate immune response which provides protection to many categories of pathogens, including viruses. It is responsible for a wide range of host responses, such as: the promotion of chemotaxis and inflammation [6], supporting the development of adaptive immunity [7, 8], opsonisation of virus particles [9, 10], neutralisation of virions and infected cells [11–13], and the enhancement of antibody-mediated neutralisation [14]. Complement proteins are predominantly synthesised in the liver and, to a lesser extent, in epithelial cells, endothelial cells, and circulating immune cells (including dendritic cells, granulocytes, macrophages, and monocytes) [14]. To regulate the activation of the complement system and to protect healthy cells, complement regulatory proteins can target various control points of the pathway. These include the cleavage of C4b and C3b (membrane cofactor protein; CD46 and factor I) [15], destabilisation of the C3 and C5 convertase (decay-accelerating factor; CD55) [16], and prevention of the MAC formation through the binding of C8/C9 proteins (protectin; CD59) [17]. Complement regulatory proteins can be expressed on the cell surface membrane of healthy cells or they can be located extracellularly in plasma.

**Fig. 1:**
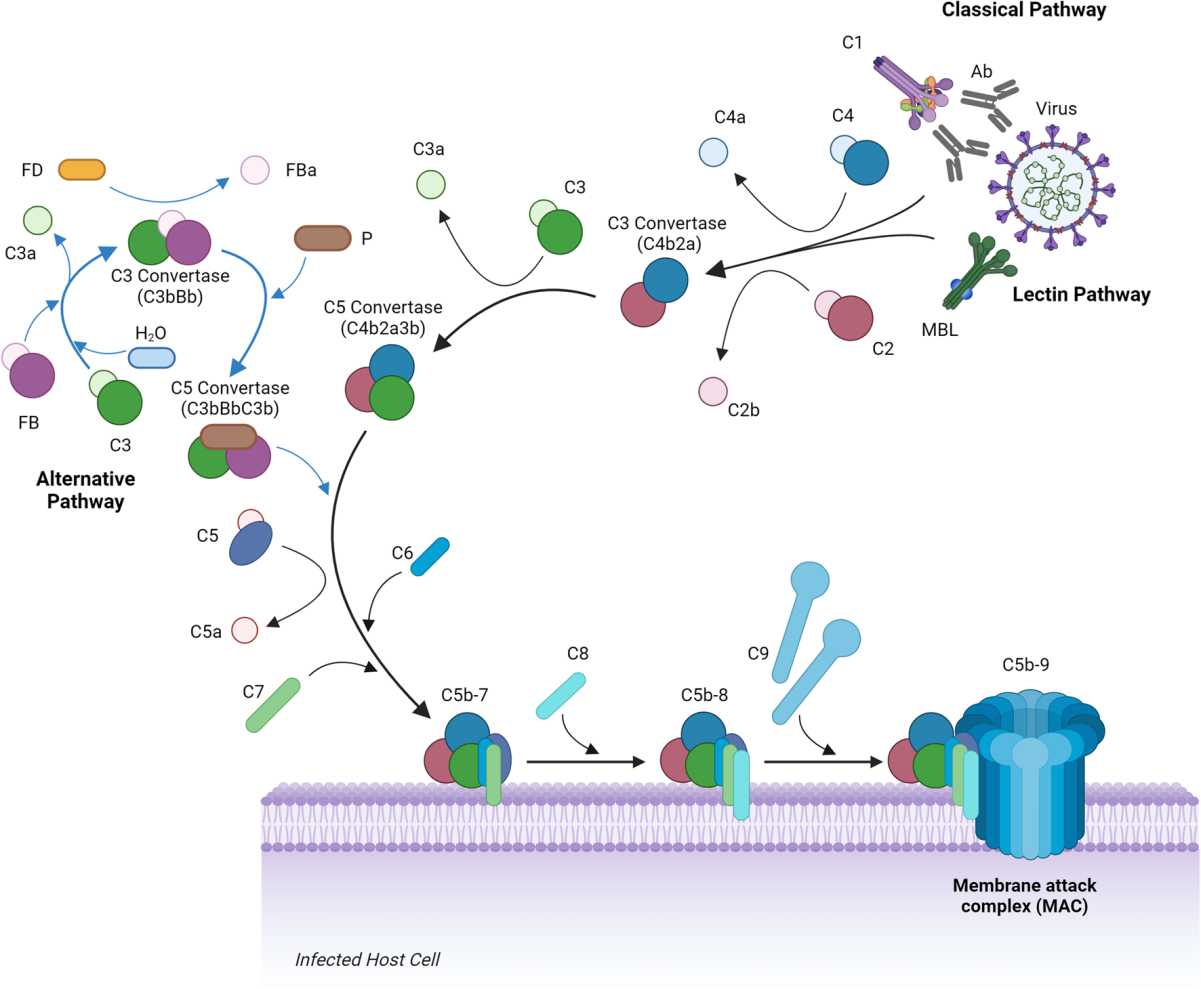
Overview of the complement system in response to viral infection, demonstrating the classical, lectin, and alternative pathways. The Figure was created using BioRender with adaptations from the “Formation of the Membrane Attack Complex” and “Life Cycle of Coronavirus” templates. Inkscape software was used to arrange the final figure. (Ab), antibody; (FB), factor B; (FD), factor D; (MAC), membrane attack complex; (MBL), mannose binding lectin; (P), properdin

The complement system can be divided into three distinct pathways: classical, lectin, and alternative (Fig. 1). The classical pathway is antibody mediated and will be the focus of discussion in this review. Typically, an antibody binds to the target antigen on the virus or virus-infected cell which allows subsequent binding of the C1q protein. C1q then forms the C1 complex with two C1r and C1s proteases, causing the enzymatic cleavage of C4 and C2 into their active components, forming the C3 convertase (C4b2a), and releasing C4a and C2b. C4b2a then cleaves C3 into C3a (an anaphylatoxin) and binds C3b to form the C5 convertase (C4b2a3b). C4b2a3b then cleaves C5 into C5a (anaphylatoxin) and binds C5b. This leads to the binding of C6, C7, C8, and multiple copies of C9, eventually forming the membrane attack complex (MAC). The C3a and C5a anaphylatoxins have broad immune regulatory functions, capable of promoting chemotaxis [6], immune cell degranulation [18, 19], the production of pro-inflammatory mediators [20], and the induction of respiratory bursts [21] through their interactions with immune cells. The lectin pathway is similar to the classical pathway, but differs in its activation. The lectin pathway is antibody independent and is activated via the binding of pattern recognition molecules (PRMs), such as mannose binding lectin (MBL), to glycosylated regions of viral antigens. MBL-associated serine proteases (MASPs) form complexes with the PRMs to cleave C4 and C2 to activate the lectin pathway [22]. The alternative pathway is typically activated via the spontaneous hydrolysis of C3 in the absence of complement regulatory proteins. The remaining C3b molecule binds factor B which is then cleaved by factor D to form the C3bBb complex. This complex is then stabilised by the binding of properdin and functions as a C5 convertase. The alternative pathway can function independently of the classical and lectin pathways, or its activation can augment both the classical and lectin pathways following their activation [14].

The significance of antibody Fc effector functions in viral infections is less commonly reported than antibody neutralisation, even though they can be of equal significance for protection. Methods of studying Fc effector functions require distinct and potentially more complex assays compared to conventional neutralisation assays, such as antibody-dependent cell-mediated cytotoxicity (ADCC); antibody-dependent cellular phagocytosis (ADCP); antibody-dependent complement deposition (ADCD); and complement-dependent cytotoxicity (CDC) assays. ADCD typically measures the deposition of C3- and C5-related products onto an antigen-coated surface or antigen-expressing cells. ADCD activity has implications for complement-mediated immune functions including inflammation, chemotaxis, opsonisation, and lysis. CDC specifically evaluates complement-mediated lysis via the induction of antigen-specific cell lysis. Of these additional Fc effector function assays the potential for complement to directly enhance antibody-mediated neutralisation remains unexplored, yet it could significantly impact neutralisation titres. Concerns over cell monlayer cytotoxicity or a lack of awareness regarding complement-mediated enhancement may explain why this phenomenon is rarely investigated. But there are methods to make this approach feasible, which will be discussed in this review.

The inclusion of complement to neutralisation assays can yield many benefits. For example, CoPs are crucial for understanding which aspects of immunity are responsible for protection against a pathogen, and to what extent a person is protected. Neutralising antibody titres are one of the primary measurements for this, but they are predominantly determined in the absence of complement which can reduce the potential for statistically significantly correlations between in vitro and in vivo findings [23–25]. Some antibodies also rely strongly on complement for complete neutralisation and in some instances neutralisation is entirely complement-dependent [26, 27]. Similarly, the early screening of antibody therapeutics, or decisions on which therapeutics maintain efficacy against new viral variants, rely strongly on neutralisation assays. Most recently, this was showcased with the emergence of SARS-CoV-2 Omicron variants which led to a drastic reduction in antibody neutralisation titres and concerns that emerging variants would escape vaccine-induced immunity. In recipients of mRNA or SARS-CoV-2 inactivated vaccines, a substantial loss of antibody neutralisation and binding to the receptor binding domain (RBD) against the Omicron variant was observed. However, binding to the full-length Omicron spike protein can remain stable along with preserved Fc-effector activity [28]. The preservation of Fc-effector activity provides an alternative means of protection to neutralisation and antibodies may retain neutralising activity when assessed in the presence of complement.

**Fig. 2:**
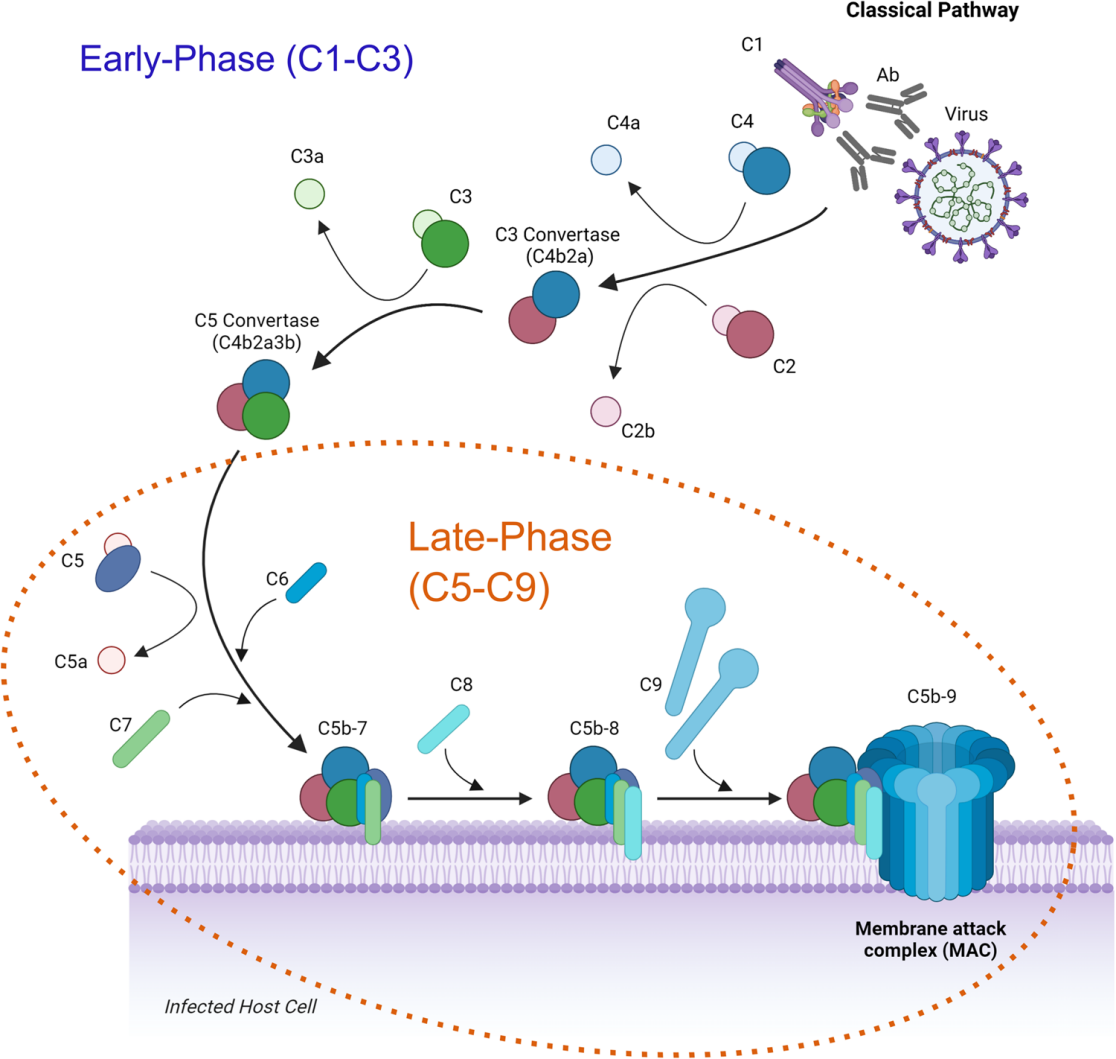
Overview of the classical pathway of the complement system in response to viral infection. For the purposes of the review, the classical pathway has been divided into two sections: the early-phase (involving complement proteins C1–C3) and the late-phase (requiring proteins C5–C9 in addition to C1–C3). (Ab), antibody; (MAC), membrane attack complex. The figure was created using BioRender with adaptations from the “Formation of the Membrane Attack Complex” template and modified using Inkscape

In this review, we will provide examples of the complement-mediated enhancement of antibody-dependent virus neutralisation, discuss the antibody characteristics which influence these interactions, explore the known underlying mechanisms for this enhancement, highlight gaps in the literature which require further research, and demonstrate where the information in this review can be applied in future research. For the purposes of this review, we have divided the complement system into two phases, the early-phase (C1–C3) and the late-phase (C5–C9), as the underlying mechanisms of enhancement can be broadly divided into these two categories, with only a few exceptions which will be highlighted throughout (Fig. 2). Typically, the early-phase relies on agglutination/aggregation of virus particles and/or prevention of cell attachment/entry, whilst the late-phase typically depends on lysis of the virus particle and/or virus-infected cell.

## Evidence for the enhancement of antibody-dependent virus neutralisation via the complement system

The potential for the complement system to enhance antibody-dependent neutralisation against many viruses has been observed through the addition of exogenous plasma as a source of complement into conventional neutralisation assays, following heat-inactivation of the immune sera or plasma. The studies discussed within this review which utilize immune sera or plasma, ensured heat-inactivation of the samples prior to the addition of an exogenous complement source. The examples discussed in this section provide evidence for complement-mediated enhancement of antibody neutralisation potency but do not investigate the underlying mechanism.

For cytomegalovirus (CMV) neutralisation, immune sera from rabbits and primates were found to contain complement-dependent neutralising antibodies [29–31]. In primates, the production of complement-dependent neutralising antibodies predominated in immune sera against human CMV (HCMV) (C87 and AD169 strains), whilst neutralising immune sera to monkey CMV (GR2598 and GR2757 strains) was complement-independent [29]. In a more recent study, vaccine-induced antibodies to CMV from rabbits also demonstrated complement-mediated enhancement. Rabbits immunized with a CRM-conjugated Antigenic Domain 2 (AD-2) peptide vaccine did not produce neutralising antibodies above the limit of detection, excluding one of the four rabbits tested, which showed a modest increase in neutralisation with the addition of rabbit complement. In contrast, all four rabbits immunised with recombinant glycoprotein B (gB) formulated with MF59 adjuvant (gB/MF59) demonstrated modest neutralising antibody titres which were all significantly enhanced with the addition of complement [32]. Underlying mechanisms for complement-mediated enhancement of CMV neutralisation have been investigated in other studies which show the importance of antigen glycosylation, antibody epitope, and antibody isotype in determining this response and are discussed later. Conformation of the antigen, the vaccine platform, and natural infection versus vaccination have also been shown to influence the immunogenicity and complement-dependency of anti-CMV antibodies. CMV-neutralising antibodies from natural infection have shown only moderate complement-mediated enhancement (two to threefold) [33], compared to a range of complement-dependent responses reported for vaccine-induced antibodies. Vaccination with trimeric recombinant HCMV gB protein, compared to the monomeric form, resulted in 11-fold higher serum titers with 50-fold higher complement-independent and 20-fold higher complement-dependent neutralising titers when using a fibroblast cell line [34]. Similarly, vaccination with recombinant gH/gL proteins in the trimeric form resulted in ~ 10-fold higher complement-dependent and complement-independent neutralising antibodies compared to the monomeric form using fibroblast and epithelial cell lines [35]. However, codelivery of gH/gL in the monomeric form using an alphavirus replicon system elicited potent complement-independent neutralising antibodies [36]. It’s unclear how complement-dependency relates to vaccine efficacy, but antigen presentation appears to profoundly influence the immunogenicity of complement-dependent and complement-independent anti-CMV responses.

For rubella virus neutralisation, the addition of guinea pig serum to plaque assays using immune sera from individuals with either recent rubella virus infections or with remote histories of rubella infection, resulted in a 4- to 16-fold increase in neutralisation titres compared to the use of heat-inactivated guinea pig serum. A heterologous antigen–antibody system was also used to deplete complement from the unheated guinea pig serum, which resulted in a loss of enhancement [37]. Similarly, the addition of guinea pig complement to varicella-zoster virus neutralisation assays resulted in a two to eightfold increase in neutralisation titres with immune sera from healthy and immunosuppressed individuals at varying periods of convalescence [38]. For influenza virus, Orthopoxviruses, and herpes simplex virus (HSV), the presence of complement can enhance antibody-mediated neutralisation [39–41] and investigations into the underlying mechanism have been described in other studies discussed later in this review.

For Ebola virus (EBOV) neutralisation, complement-mediated enhancement of neutralising antibodies was first identified by Wilson et al. [23]. Murine monoclonal antibodies were classified into five groups depending on their performance in neutralisation assays and therapeutic studies using mice infected with mouse-adapted EBOV. The ability of the monoclonal antibodies to neutralise EBOV in vitro did not always translate to the in vivo findings. However, when complement was added to the neutralisation assays, the monoclonal antibodies in two of the classified groups were then able to neutralise EBOV in concordance with their performance in vivo. All of the antibodies which provided complete protection were of the IgG2a subclass, which is the most potent complement activating subclass in mice. In a later study by Rimoin et al. [42] using EBOV disease (EVD) convalescent plasma collected 40 years post-infection, the addition of guinea pig complement did not enhance neutralisation titres. However, a subsequent study by Mellors et al. [43] using EVD convalescent plasma from individuals with more recent EBOV infections found that the addition of pooled human plasma as an exogenous complement source could enhance the neutralisation of wild-type EBOV. All samples were capable of mediating ADCD in vitro, but the enhancement of neutralisation was only observed for some plasma samples. An exact mechanism for this enhancement remains to be determined. Where CoPs remain undetermined, the translation of findings in vitro to in vivo are extremely important. For EBOV in particular, antibody binding and Fc-effector functions, in addition to neutralisation titres, all show a strong relationship with protection [44]. Further examples are discussed later in this review where the addition of complement to neutralisation assays strengthened the relationship between in vitro and in vivo findings in support of a CoP.

Early evidence for the complement-mediated enhancement of antibody-dependent Hantavirus neutralisation was demonstrated using sera from convalescent patients with haemorrhagic fever with renal syndrome (HFRS) against Hantaan virus in plaque reduction neutralisation tests (PRNTs). The addition of guinea pig complement to immune sera from humans in PRNTs resulted in two- to thirty-six-times higher neutralisation titres. Similarly, the addition of guinea pig complement to immune sera from rats was shown to enhance neutralisation titres (≤ 35-fold) and neutralisation was almost entirely dependent on the presence of complement [45]. Later studies made similar observations, providing further evidence for the role of complement in the enhancement of Hantavirus neutralisation. Asada et al. [46] observed that anti-Puumala virus immune sera from mice was dependent on complement for cross-reactive neutralisation of Hantaan virus*,* when supplementing with rabbit serum at a final concentration of 3% as a source of complement. However, no cross-reactive neutralising activity was observed in the absence of complement. Only low levels of cross-reactive antibody binding to Hantaan virus were detected. In vivo, the transfer of anti-Puumala virus immune serum demonstrated cross-protection against Hantaan virus, which could support the in vitro findings for complement-dependent neutralising activity, although it does not rule out the possibility of other antibody immune effector functions required for protection. The same methodology was applied using anti-Prospect Hill virus immune sera but no cross-protection was observed against Hantaan virus, irrespective of complement. Similar to the anti-Puumala virus immune sera cross-reactivity, antibody binding to Hantaan virus was low. The importance of complement for cross-reactive antibody-mediated neutralisation could be a consideration for other viruses where emerging variants are a concern and therapeutic options are limited, such as SARS-CoV-2.

Further evidence for the role of complement in Hantavirus neutralisation comes from Hooper et al. [47]*.* They noticed a drastic decrease in PRNTs against Seoul virus following the heat inactivation of vaccine-induced immune sera from BALB/c mice and Syrian hamsters. As complement is a heat-labile component of sera, they replenished the complement system through supplementation with guinea pig complement at a final concentration of 5%. PRNT titres then increased 4- to 32-fold, whilst the guinea pig complement alone did not have an effect on PRNT. In more recent studies, complement is often a common component of Hantavirus neutralisation assays [48, 49]. Despite the addition of complement being more common place in Hantavirus neutralisation assays, there is little speculation regarding the underlying mechanism of action for its enhancement of neutralisation.

The examples discussed in this section demonstrate the significant implications of the complement system for antibody-dependent neutralisation, independent of cell-mediated immunity. This shows a direct implication for understanding neutralisation in both an in vitro and in vivo context. To further evaluate its significance, it is important to understand the underlying mechanism and which factors are responsible for this phenomenon.

## Antibody characteristics which influence complement activation

This review focuses on the antibody-dependent classical pathway of the complement system, and so antibody characteristics can be important determinants of complement activity. Such characteristics include: epitope specificity, glycosylation of both the antigen and antibody, and antibody isotypes. The relative concentrations of antibodies and complement components can also influence complement activity.

### Epitope specificity

Epitope specificity can influence the level of complement activation via the classical pathway. Conventional activation of this pathway requires antibody binding, followed by the binding of C1q to IgM or multiple IgG molecules. For C1q to bind efficiently and with high avidity to IgG antibodies, the formation of hexameric IgG structures are required through the interactions with neighbouring IgG molecules [50]. This clustering of IgG molecules will, therefore, be influenced by the antibody epitopes and their density and distribution. Epitope density also impacts IgG subclasses differently, with IgG1 demonstrating the most efficient complement activation at high epitope densities, and IgG3 being most efficient at low epitope densities in some instances [51, 52]. The effect of antibody subclasses on complement activation is later described in more detail.

Cranage et al. [53] first demonstrated the importance of epitope specificity for complement-mediated neutralisation of HCMV. A gH-specific murine monoclonal antibody showed complement-independent neutralising activity, whilst five monoclonal antibodies with specificity for anti-HCMV gB were dependent on complement for virus neutralisation. Li et al. [54] also demonstrated the importance of epitope specificity for complement-mediated neutralisation of HCMV. Three non-neutralising rabbit monoclonal antibodies against the recombinant gB, with varying epitope specificity, were used to investigate the mechanism behind prior work on complement-dependent HCMV neutralisation [32]. Two of the three monoclonal antibodies were capable of neutralisation when rabbit complement was supplemented into the assays. Despite this difference in complement-dependence, all antibodies were of the same isotype. Neutralisation was also dependent on administration prior to attachment and entry, and no cytotoxicity was observed, suggesting the targeting of cell-free virions. Neutralisation titres did not correlate with affinity either, supporting the hypothesis that epitope specificity determined the complement-mediated enhancement. In one final observation, the potency of complement-mediated neutralisation also varied between cell types. Using paired plasma from individuals vaccinated during Phase I gB/MF59 trials, a 15-fold increase in neutralisation titre was observed with the addition of complement in MRC-5 cells, but only a 1.6-fold increase with ARPE-19 cells. The authors speculate that this difference may also be due to epitope specificity, as certain regions may be more effective at blocking fibroblast entry than epithelial entry [54].

For EBOV neutralisation, Wilson et al. [23] showed that antibodies directed towards five unique epitopes were protective in mice in vivo, but did not show efficacy in PRNTs. In the presence of complement, plaque reduction was observed for some antibodies which were all of the IgG2a subclass. Interestingly, antibodies of the same subclass which recognized different epitopes did not all show an increase in neutralisation when complement was added in vitro, which suggests that both the epitope and antibody subclass were important determinants of this response. Feng et al. [24] made similar observations for the complement-mediated enhancement of influenza virus, where both antibody epitope and isotype influenced the extent of complements contribution to haemagglutinin (HA) inhibition. They also note that greater enhancement was observed with primary versus secondary antibody responses. The basis for this is unclear, but could be influenced by antibody characteristics such as affinity/avidity or epitope specificity.

### Glycosylation

Glycosylation of both the antigen and the antibody are important considerations for complement-mediated enhancement. Antibody glycosylation affects Fc-mediated functions of the wider humoral immunity in a complement-dependent manner against viral infections such as HIV [55, 56], and can directly impact C1q binding and complement activation depending on the levels of galactosylation and sialylation on the Fc region [57, 58]. Glycosylation of the antigen can also influence antibody-dependent neutralisation. In a study by Britt et al. [59], recombinant HCMV protein gp55-116 (an earlier designation for gB) was expressed in *E. coli* and in mammalian cells infected with a recombinant vaccinia virus (VACV), which were then used to immunise mice. Immunisation with VACV recombinant GP55-116 almost exclusively produced complement-dependent neutralising antibodies, whereas the use of *E. coli* derived protein resulted in a significantly higher amount of complement-independent neutralising antibodies, despite significantly lower overall antibody titres. The authors suggest that this difference may be due to the absence of glycosylation on the *E. coli*-derived antigen.

### IgG subclasses

Once an antibody has bound its target, the Fc region is an important determinant of C1q binding and complement activation. Typically, IgG1 and IgG3 are the most efficient at binding C1q and activating the complement system, IgG2 shows low level complement activation, and IgG4 does not activate the complement system. However, there are some exceptions to this order. For example, whilst IgG3 has been shown to bind more C1q molecules than IgG1, IgG1 was more efficient in mediating complement-dependent lysis [60]. IgG4 does not bind C1q [51, 52, 60–62] but does reportedly show some level of complement activation under specific conditions [51, 60], although other studies report no complement activity and suggest its function may even be inhibitory [52, 63]. The order of IgG subclasses relative to complement activation also varies depending on epitope density and distribution, as mentioned previously [51, 52].

In a study by Cohen et al. [64], antibody isotype was important for the complement-mediated antibody neutralisation of extracellular enveloped virion (EEV) VACV particles. Anti-A33 and anti-B5 antibodies neutralised VACV by lysis and opsonisation of the virus particles, respectively. The difference in mechanism was associated with the concentration of bound antibody and the IgG2a complement-activating subclass in mice correlated with protection. Similarly, Benhnia et al. [65] showed that the only potent neutraliser in a panel of B5 murine monoclonal antibodies was of the IgG2a subclass, and that protection in vitro and in vivo was dependent on complement.

## Mechanisms underlying the complement-mediated enhancement of antibody-dependent neutralisation

Once an antibody has bound to the viral antigen and the complement cascade has been initiated, the mechanism for complement-mediated enhancement of antibody-dependent neutralisation can roughly be divided into two broad categories: the early-phase (C1–C3) and the late-phase (C5–C9).

**Fig. 3:**
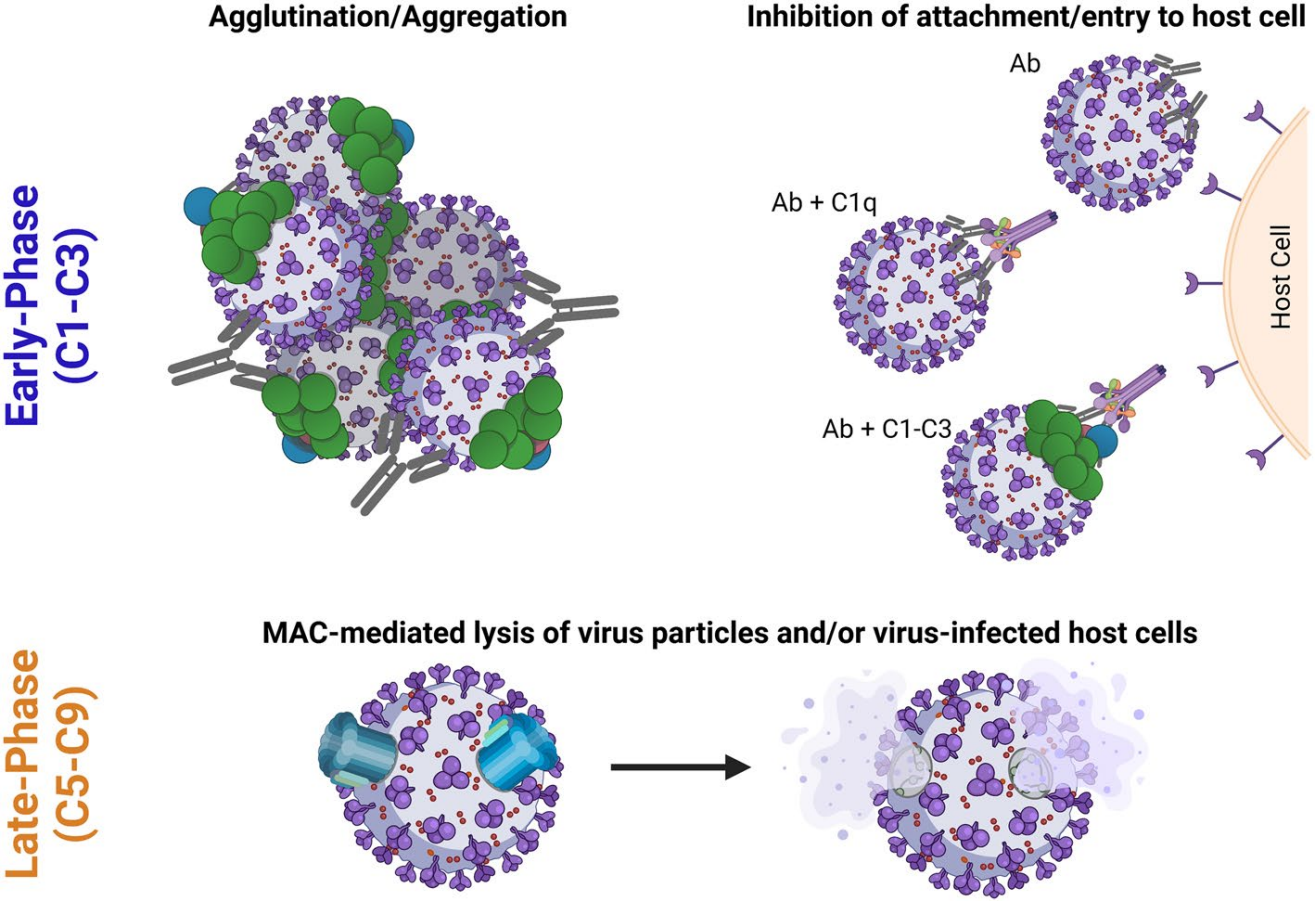
Mechanisms underlying the complement-mediated enhancement of antibody-dependent virus neutralisation. Agglutination/Aggregation: Complement deposition (figure depicts C4 [blue circles], and C3 [green circles]) can lead to the agglutination/aggregation of virus particles to enhance antibody-mediated neutralisation. Inhibition of attachment/entry to host cells: C1q binding and subsequent complement deposition can enhance antibody-mediated neutralisation by coating virus particles and blocking virus attachment/entry. MAC-mediated lysis of virus particles and/or virus-infected host cells: Formation of the membrane attack complex (MAC) on virus particles and/or virus-infected cells expressing viral antigens can induce lysis to enhance neutralisation. This figure was made using BioRender with adaptations of the “Formation of the Membrane Attack Complex” and “SARS-CoV-2, 2 Panels (Layout 1 × 2)” templates

Enhanced neutralisation with complement proteins C1–C3 typically occurs via agglutination/aggregation of virus particles or the inhibition of viral attachment and/or entry to the host cell. When complement components C5–C9 are required for neutralisation, the mechanism is often lysis of the virion and/or infected host cells (Fig. 3). There are some exceptions to this rule i.e., C5-dependent neutralisation of HSV which is not dependent on lysis. More than one of these mechanisms may also occur simultaneously or be dictated by relative protein concentrations. Further mechanisms may also exist that are currently undefined. In this section, the studies which have demonstrated an underlying mechanism for complement-mediated enhancement of antibody-dependent virus neutralisation have been subdivided into the early-phase and late-phase. A summary of all viruses discussed within this review, and the corresponding studies, are listed in Table 1.

**Table 1:** A summary of all the viruses discussed within this review and the corresponding publications which show a complement-mediated enhancement of antibody neutralisation titres

Study overview: complement-mediated enhancement of neutralisation titres				
Virus	Factors involved in neutralisation	Study summary	Reported enhancement	References
Avian infectious bronchitis virus	IgG mediated? Not specified IgM Mediated? Not specified C1**–**C3 Mediated? Not specified C5**–**C9 Mediated? Not specified	Electron microscopy revealed viral lysis (suggesting a C5–C9 dependent mechanism) following treatment of avian infectious bronchitis virus with unheated serum. This effect was abolished with heat-inactivation and could be replenished with the addition of guinea pig or rabbit complement, which suggests that this activity was complement-mediated	Complement enhanced the neutralisation potency of immune sera by ~ 2 log. Units	[66]
Cytomegalovirus	IgG mediated? Yes IgM Mediated? Yes C1-C3 Mediated? Not specified C5-C9 Mediated? Not specified	Epitope specificity, glycosylation of target antigen, and the Fc region were shown to be important determinants of complement-mediated activity. One study showed that the enhancement of neutralisation was lost once viral attachment and cell entry had occurred	Complement enhanced the neutralisation potency of immune sera by ≤ 20-fold Complement was essential for neutralisation with some purified human, rabbit, and murine antibodies, and with rabbit sera	[31, 33–36, 53, 54, 59, 67, 68]
Ebola virus	IgG mediated? Yes IgM Mediated? Untested C1**–**C3 Mediated? Not specified C5**–**C9 Mediated? Not specified	Enhancement has been demonstrated with convalescent human sera and purified murine monoclonal IgG antibodies. Antibody epitope and isotype were both shown to be of importance in determining this response	Complement enhanced the neutralisation potency of immune sera by < 2.5-fold Complement was essential for neutralisation with purified IgG	[23, 43]
Epstein-Barr virus	IgG mediated? Yes IgM mediated? Untested C1**–**C3 Mediated? Yes C5**–**C9 Mediated? No (C8-independent)	Use of purified C1–C3 proteins with purified IgG was sufficient for neutralisation, whilst the use of C8-depleted plasma did not impact neutralisation. No viral aggregates were observed either, suggesting that interference with cell attachment/entry was the likely mechanism of neutralisation	Complement was essential for neutralisation with purified IgG	[69]
Equine arteritis virus	IgG mediated? Yes IgM Mediated? Untested C1-C3 Mediated? Yes C5-C9 Mediated? Yes (in low antibody concentrations)	At high antibody concentrations, proteins C1-C3 were sufficient to enhance neutralisation. At low antibody concentrations, proteins C5-C9 were required and induced lysis of the virus particles	Complement was essential for neutralisation with purified IgG	[70]
Hantaviruses	IgG mediated? Not specified IgM mediated? Not specified C1**–**C3 mediated? Not specified C5**–**C9 mediated? Not specified	Neutralisation with human, mice, guinea pig, and rat sera was enhanced with complement. In some instances, complement was essential for neutralisation. Complement was also required for antibody-mediated cross-protection in some studies	Complement enhanced the neutralisation potency of immune sera by 1.7 to 32-fold (human sera) and ≤ 35-fold (rat sera) Complement was essential for neutralisation with cross-reactive immune sera	[45–49]
Herpes simplex virus-1/2	IgG mediated? Untested IgM mediated? Yes C1**–**C3 Mediated? Yes C5**–**C9 Mediated? Partially	Complement proteins C1–C3 were required for neutralisation with IgM, dependent on their relative concentrations. C5 was required for enhancement in some studies, but not others. Where C5 was required, the mechanism was not agglutination, lysis, or interreference with cell attachment. Instead, the stages from cell entry to early viral gene expression may have been impacted	Complement was essential for neutralisation with purified IgM	[71–73]
Influenza Virus	IgG Mediated? Yes IgM Mediated? Yes C1-C3 Mediated? Yes C5-C9 Mediated? Yes	In some instances, the addition of C1q was sufficient to enhance neutralisation. Other studies show the C1–C3 proteins were required for neutralisation with nIgM and IgG, which resulted in the aggregation of virus particles. The presence of complement also enabled cross-reactive neutralisation of virus-infected cells via lysis	Complement was essential for neutralisation with some human immune sera and purified nIgM Complement enhanced the neutralisation potency of purified murine IgG by 10- to 100-fold	[12, 24, 25, 74]
Newcastle disease virus	IgG mediated? Yes IgM Mediated? Yes C1-C3 Mediated? Yes C5-C9 Mediated? No	Collection of serum post-vaccination was associated with complement dependency. Both IgG and IgM fractions were complement-dependent and components C1-C3 were sufficient for neutralisation with IgM	Complement was essential for neutralisation with purified IgG and IgM	[26]
Polyoma virus	IgG mediated? Yes IgM mediated? Untested C1**–**C3 mediated? Yes C5**–**C9 mediated? No (C6-independent)	The complement-mediated enhancement of neutralisation was only observed in low antibody concentrations. Enhancement was C3-dependent and resulted in the aggregation of virus particles. No lysis was observed and C6-deficient sera did not impact neutralisation	Complement enhanced the neutralisation potency of purified IgG by > 80%	[27]
Rubella virus	IgG mediated? Not specified IgM Mediated? Not specified C1**–**C3 Mediated? Not specified C5**–**C9 Mediated? Not specified	Enhancement of neutralisation was demonstrated with the addition of guinea pig complement to sera from individuals with recent and remote histories of infection	Complement enhanced the neutralisation potency of immune sera by 4- to 16-fold	[37]
Simian immunodeficiency virus	IgG mediated? Not specified IgM Mediated? Not specified C1**–**C3 Mediated? Not specified C5**–**C9 Mediated? Not specified	The complement system was required for the sterilising protection of vaccinated macaques. The titre of complement-dependent anti-HLA antibodies was associated with protection in vivo	Complement was essential for neutralisation with immune sera from some vaccinated macaques	[75]
Vaccinia virus	IgG mediated? Yes IgM Mediated? Not specified C1-C3 Mediated? Yes C5**–**C9 Mediated? Yes (sometimes)	Enhancement of neutralisation, and whether it is C1–C3 or C5–C9 mediated, typically depends on the concentration of bound antibody. A unique two-step process of complement-mediated enhancement of neutralisation has been proposed, whereby the EEV membrane is lysed and the IMV is neutralised by a secondary antibody. The addition of complement in vitro leads to better correlations with in vivo results	Complement enhanced the neutralisation potency of immune sera by > 10-fold in low antibody concentrations Complement was essential for neutralisation with purified IgG	[64, 65, 76, 77]
Varicella-Zoster virus	IgG mediated? Not specified IgM Mediated? Not specified C1**–**C3 Mediated? Not specified C5**–**C9 Mediated? Not specified	Enhancement was demonstrated with the addition of guinea pig complement to immune sera shortly after acute infection with varying periods after convalescence	Complement enhanced the neutralisation potency of immune sera by two to eightfold	[38]
Vesicular Stomatitis Virus	IgG mediated? Yes IgM Mediated? Yes C1**–**C3 Mediated? Yes C5**–**C9 Mediated? No	Enhancement of neutralisation only required proteins C1–C3. In one study, this effect was lost with high concentrations of antisera. In another study using purified IgM, neutralisation was entirely complement-dependent	Complement enhanced the neutralisation potency of immune sera by > 10-fold in low antibody concentrations Complement was essential for neutralisation with purified IgM	[76, 78]

The studies are briefly summarised for each virus including the reported level of enhancement. “Not specified” = may have been indirectly tested but its precise involvement cannot be determined. “Untested” = excluded from the experiment

### Early-phase (C1–C3)

The early-phase complement proteins (C1–C3) can directly influence antibody neutralisation through several mechanisms. Firstly, the deposition of complement proteins onto virus particles can prevent their interactions with host cell receptors to inhibit entry and attachment. This effect is greater than antibody binding alone, with up to 1000 C3b molecules capable of binding within the vicinity of a single C3 convertase [79]. Secondly, complement deposition can cause virion aggregation/agglutination to promote antibody neutralisation. The relative concentrations of antibodies and complement proteins can influence this activity.

For influenza virus, the binding of antibody and C1q protein alone can be sufficient to enhance neutralisation. HA-specific monoclonal antibodies with poor neutralising activity in vitro showed enhanced neutralisation in the presence of complement, which corresponded to improved neutralisation in vivo. The epitope, isotype, and whether it was a primary or secondary antibody response influenced the extent of enhancement with complement. Enhancement was predominantly observed with murine IgG2a and IgG2b antibodies from the primary response and the enhancement with complement was reproduced with purified C1q-alone. The authors proposed that enhancement was primarily mediated via improved steric inhibition with the addition of C1q and, to a lesser extent, C1q-mediated the stabilization of low avidity IgG-virus complexes [24].

In a study by Beebe et al. [74], complement components C1–C3 were required for the enhanced neutralisation of influenza virus with IgG. Complement proteins C4 and C3 were deposited on the virion surface and neutralisation could occur in the absence of lysis. Jayasekera et al. [25] observed that neutralisation of influenza A virus (IAV) with purified natural (nIgM) was also dependent on complement components C1–C3. In vitro studies with mouse-adapted IAV and pooled mouse serum showed concentration-dependent neutralisation with serum and this effect was lost following heat-inactivation or depletion of secretory IgM, C1q, C4, or C3. However, the use of C5-deficient serum did not impact neutralisation, which suggests a mechanism other than lysis. This was supported with the use of electron microscopy, which revealed that the presence of nIgM and complement resulted in the aggregation of virus particles. Further investigations in vivo showed that nIgM was partially protective in RAG1^−/−^ mice during the early-phase of IAV infection. The translation between in vitro and in vivo findings in this example is better represented with the inclusion of complement, as nIgM did not neutralise in absence of complement in vitro, but demonstrated partial protection in vivo. In a study by Beebe and Cooper [78], purified nIgM from human serum exhibited complement-dependent neutralisation of VSV with the use of purified complement proteins C1–C3. Neutralisation of VSV was not achieved with the use of nIgM or complement proteins C1–C3 individually, but their combined use led to C3 deposition and virus neutralisation.

The relative concentrations of antibody and complement proteins have been shown to influence complement-mediated neutralisation for several viruses. For VACV and vesicular stomatitis virus (VSV), the addition of early-phase complement proteins (C1–C3) was required for neutralisation with antisera from rabbits and humans. C5- and C6-deficient sera did not impact neutralisation and the effect was heat labile. However, the enhancement with C1–C3 was abrogated with the use of higher antibody concentrations [76]. Similarly, the neutralisation of polyoma virus with low antibody concentrations was shown to be C3-dependent with the use of sera depleted in various complement proteins. C6-deficient complement did not impact neutralisation, whereas the enhancement of neutralisation was abolished with the use of C4- or C2-deficient complement. Using purified proteins C1–C3, C3 was shown to be essential for enhancement and resulted in viral aggregation. This effect was lost with the use of high antibody concentrations [27]. The authors note that whilst they did not observe viral lysis, the terminal complement proteins were consumed in the process. The lack of lysis is perhaps unsurprising as polyoma virus is a non-enveloped virus and the MAC formation requires a lipid membrane. The consumption of terminal complement proteins may, therefore, be a by-product of activation.

Some studies have shown a change over time in the complement-dependence of sera for neutralisation. In a study by Linscott and Levinson [26], rabbits were immunised with Newcastle disease virus and only the sera collected 6 days post-infection showed complement-dependent neutralisation, whereas the sera collected 2-, 4-, and 9-weeks post-infection showed complement-independent neutralisation. Both the IgM and IgG fractions of the 6 days post-infection sera were isolated and shown to be complement dependent. Further investigations with the IgM fraction showed that only the addition of complement components C1–C3 was required for this enhancement. Further investigations into the IgG fraction were not reported. The authors speculated that the complement-dependence may be related to antibody affinity, as dependence decreases with time post-infection as the result of affinity maturation. High antibody affinity can correlate with neutralisation potency, and so low affinity antibodies may rely on other mechanisms for neutralisation such as complement-mediated agglutination or the lysis of virus particles. Consequently, the complement-enhancing effect may be most apparent for antibodies with low neutralisation potency and low affinity. However, class-switching and a waning of IgM concentrations in the later timepoints could be a factor in this study as well. The authors also add that whilst the addition of C1–C3 was sufficient for enhanced neutralisation, a further additive effect might be observed with the addition of complement proteins C5–C9 [26]. This has been demonstrated for the neutralisation of equine arteritis virus (EAV). A purified IgG preparation following experimental infection of a horse with EAV showed enhanced neutralisation with purified complement proteins. When the IgG was in excess, high concentrations of C1–C3 were sufficient for complete neutralisation. Whilst the addition of C5–C9 resulted in lysis of the virion in these conditions, it did not enhance neutralisation. However, when the IgG concentration was low, components C5–C9 enhanced neutralisation by means of lysis [70].

Daniels et al. [71] showed that a complement-mediated enhancement of HSV neutralisation with IgM was dependent on both antibody and relative complement protein concentrations. The use of IgM and C1 protein was not sufficient for neutralisation. The addition of C4 in excess resulted in neutralisation, without the need for C2 and C3. When the concentrations of C4 were no longer in excess, C2 was then required for optimal neutralisation. And when C2 was no longer in excess, C3 was required to achieve optimal neutralisation. Finally, in low concentrations of IgM, the use of whole guinea pig complement resulted in higher neutralisation than the individual components. The use of C5- and C6-deficient complement did not impact neutralisation, and so formation of the MAC was not responsible for this enhancement. Because complement proteins C1–C3 in various combinations were sufficient to neutralise HSV, neutralisation may occur via agglutination and/or prevention of cell entry/attachment via complement deposition.

Interestingly, other studies have demonstrated a critical role of C5 in the neutralisation of HSV, but not the remaining terminal complement proteins (C6–C9). Hook et al. [73] demonstrated that natural IgM (nIgM) from non-immune human serum, which is present without prior antigenic exposure unlike immune/adaptive IgM, exhibited complement-dependent neutralisation of HSV-1 and HSV-2 gC-null viruses. Wild-type HSV strains were resistant to complement-mediated neutralisation via the gC protein, and such evasion mechanisms of viruses have been reviewed previously [14, 80, 81]. Using plasma depleted in various complement proteins, neutralisation was shown to be dependent on C1q, C3, and C5. However, depletion of C6 did not impact neutralisation [73]. This mechanism was also independent of C8 and factor D for the neutralisation of HSV-1 gC-null virus [72]. Neutralisation of HSV-1 and HSV-2 gC-null viruses did not rely on lysis, virion aggregation, nor interruption of cell attachment. However, early viral gene expression was inhibited. The authors, therefore, concluded that the complement-dependent neutralisation could affect cell entry, uncoating, translocation of viral DNA to the nucleus, or initiation of early viral gene expression [72, 73]. Whilst the complement system does have several antiviral intracellular functions, this is typically reported for non-enveloped viruses as complement deposits remain on the virion surface following cell entry, as opposed to the lipid membrane of enveloped viruses which is removed [14]. Therefore, a mechanism affecting cell entry and/or uncoating may be the most likely explanation for this enveloped virus. Whether the C5 protein was required for the enhancement of HSV neutralisation titres may depend on whether the proteins C1–C3 were in excess in previous studies. Other factors which could account for variations between studies of complement-mediated enhancement of neutralisation titres, include: the antibody characteristics as discussed earlier; the use of different viral strains; the complement source i.e., rabbit, guinea pig, human, or purified proteins; and the use of purified antibodies versus plasma or serum.

The addition of early-phase complement proteins can also promote cross-reactive neutralisation, which has been demonstrated for Epstein-Barr virus (EBV) with an HSV-1 antibody. EBV neutralisation with non-EBV immune sera could be attained with the addition of complement components C1–C3. This response was IgG-dependent and complement-dependent with exception of the C8 protein. There was no evidence of viral aggregation or disruption as determined via gradient ultracentrifugation, which suggests neutralisation was not mediated by virion aggregation or the MAC and lysis. A possible mechanism could, therefore, be the prevention of cell attachment and entry via complement deposition [69].

The mechanisms described within this section demonstrate that complement-mediated enhancement by the early-phase proteins (C1–C3) primarily depends on virion aggregation/agglutination or inhibition of cell entry/attachment. There are exceptions to this rule, as discussed for HSV-1 and HSV-2, and it is possible that other mechanisms also exist. For example, the combination of antibodies and complement proteins can induce a potent intracellular antiviral response against non-enveloped viruses [82]. Whilst this has not been explicitly described to enhance neutralisation titres, it could be a potential mechanism.

### Late-phase (C5–C9)

Enhancement of antibody-dependent neutralisation via the complement system that requires the late-phase proteins (C5–C9), typically occurs via lysis of virions and/or host cells. The virus structure can influence this response as a lipid membrane, such as those on enveloped viruses, is required for complete formation of the MAC [83, 84].

The requirement of late-phase proteins versus early-phase proteins for enhanced neutralisation is less often reported. This might be partly attributed to the early-phase proteins often being sufficient for neutralisation when tested in excess. One example of this is the complement-mediated enhancement of EAV neutralisation discussed previously; EAV neutralisation with high concentrations of purified IgG was enhanced with the addition of complement proteins C1–C3. Whilst lysis of the virions occurred, it did not contribute to neutralisation. However, when the IgG concentration was low, complement proteins C5–C9 were then required for enhancement which resulted in lysis of the virus particles [70]. The MAC was also proposed to be an active component in the neutralisation of avian infectious bronchitis virus (IBV), as viral lysis was observed following treatment with unheated serum compared to heated serum [66]. Other complementary effectors may be required for the MAC to induce lysis, which could further explain why this mechanism is less often reported. Firstly, the assembly of multiple MACs may be required for the lysis of nucleated cells [85]. Secondly, various cell types are capable of shedding plasma membrane-inserted MACs by endocytosis or vesiculation to protect against complement-mediated lysis [86]. Lastly, the mechanism for lysis can be a combination of factors and precise mechanisms are often unclear, but can include osmotic deregulation and the induction of apoptosis for some pathogens and nucleated cells [87–89]. There is a paucity of studies describing the complement-mediated mechanism for lysis of virus particles, despite evidence via electron microscopy [11].

The relative concentrations of antibody and complement proteins vary between individuals and can be influenced by immune evasion mechanisms. In a study by Cohen et al. [64], anti-B5 sera neutralised the extracellular virions (EV) of VACV by opsonisation or viral lysis, dependent on the concentration of bound antibody. EV particles can incorporate host cell complement regulatory proteins CD55 (inhibitor of C3 and C5 convertase formation) and CD59 (inhibitor of MAC formation) into the virus particle as an acquired complement regulatory mechanism. At high concentrations of anti-B5 sera, CD55-expressing virions were partially protected from complement-mediated neutralisation whereas CD59-expressing virions were not protected. However, at low anti-B5 concentrations, expression of CD55 or CD59 provided equal protection, suggesting a mechanistic switch from opsonisation to viral lysis dependent on antibody concentration.

The earlier discussion of influenza viruses showed that neutralisation with nIgM was dependent on complement components C1–C3, which resulted in the aggregation of virus particles [25]. IgG also demonstrated complement-dependent neutralisation via the deposition of C4 and C3 protein, in the absence of viral lysis [74]. However, in a study by Terajima et al. [12], a more critical role of the MAC was described. HA-specific monoclonal antibodies cloned from plasmablasts of patients infected with 2009 pandemic influenza, or recipients of pre-pandemic seasonal influenza vaccines, were characterised by complement-dependent lysis assay. Whilst the majority of the antibodies were neutralising, only some could mediate lysis. Two of the three monoclonal antibodies which bound to the stalk region of the HA molecule could lyse both the virus particles and virus-infected cells. These two antibodies also demonstrated greater cross-reactivity to distant H1N1 strains compared to the other neutralising antibodies. One of these antibodies was also cross-reactive between H1 and H2 subtypes, demonstrating that complement may enhance cross-reactivity as well as neutralisation titre.

A novel, two-step complement-mediated mechanism of VACV neutralisation has been proposed, which is partially dependent on lysis. The EV of VACV exists as an extracellular enveloped virion (EEV) and a cell-associated enveloped virion (CEV). Lustig et al. [77] showed that both forms of the EV are susceptible to complement-mediated neutralisation. Firstly, an antibody to the viral A33 protein of the outer membrane could bind and initiate complement-dependent lysis to expose the intracellular mature virion (IMV). Secondly, an antibody to the viral L1 protein of the released IMV could then bind and neutralise. It was mentioned previously that Benhnia et al. [65] identified a potent B5 neutralising monoclonal antibody of the IgG2a subclass against VACV, which required complement for complete protection in vitro and in vivo. Further investigations showed several mechanisms for this. Firstly, neutralisation of free virions was C5-independent and likely occurred via agglutination. Secondly, complement-mediated lysis of infected cells which expressed the viral B5 protein contributed to a reduction in viral titres. Lastly, in vivo, other Fc effector functions were important for protection. The authors noted that the neutralisation assays in the presence of complement were a better predictor of in vivo efficacy compared to conventional neutralisation assays and comet tail inhibition assays. Only 1/8 monoclonal antibodies provided protection in vivo. In vitro results from the complement neutralisation assays corresponded completely with these results (*n* = 8), whilst conventional neutralisation assays predicted that none of the monoclonal antibodies would provide protection (*n* = 8) and the comet tail inhibition assays predicted that all monoclonal antibodies would provide protection (*n* = 3). The complement system was also required for the sterilising protection of vaccinated macaques against simian immunodeficiency virus (SIV). Complement-mediated neutralisation of SIV by antibodies against HLA proteins incorporated into the virion during the budding process, correlated with the sterilising protection of SIV-vaccinated macaques. The ability to differentiate protected from unprotected macaques in vivo was dependent on the titre of complement dependent, rather than complement independent, neutralising antibodies in vitro. The titres of these complement-dependent antibodies were not remarkably high and previous evidence suggests that the protection could be via complement-mediated lysis of the virus particles [75, 90].

## Considerations and future applications

The complement system can have significant implications for determining neutralisation titres in vitro. Conventional neutralisation assays often exclude the functional activity of the complement system through heat-inactivation of sera/plasma samples or the use of certain anticoagulants, which can subsequently impact interpretations of CoPs, therapeutic efficacies, and vaccine responses. Similarly, the approach to not heat-inactivate sera/plasma samples can lead to variability within neutralisation assays, as the remaining complement activity within sera/plasma samples can vary. Therefore, a common approach to studying complement-mediated enhancement of neutralisation is to heat-inactivate the immune sera/plasma, and use exogenous sera/plasma or purified proteins to uniformly restore the complement system to provide consistent results.

There are a number of practical considerations for utilising complement in neutralisation assays which can add to the overall complexity. These practical considerations include: possible complement-mediated cytotoxicity; the cell line used for virus infection and propagation; and the complement source. One of the primary reasons for heat-inactivating samples prior to their use in neutralisation assays is to alleviate concerns of cytotoxicity. The extent of complement-induced cytotoxicity can vary between sera/plasma samples and cell lines, so this would need to be considered for each series of experiments. Heat-inactivation of the immune sera and supplementation with exogenous sera/plasma as a complement source allows the concentration of complement to be consistent, controlled, and individually assessed prior to the experiment. Many of the studies discussed within this review demonstrate enhancement using relatively low concentrations of exogenous sera/plasma (< 5%), further minimising the risk of cytotoxicity. The concentration of complement proteins varies between individuals (influenced by factors such as age, gender, and genetics [91–93]) and so pooling the sera/plasma samples from multiple donors can negate the effects of complement irregularities to ensure better consistency. Antibodies against the pathogen of interest in the complement source may require immunodepletion, as described in published methods for the standardised depletion of IgG and IgM in pooled human plasma [94]. It is also recommended that freeze–thaw cycles are avoided, although the reported effects of this on complement activity can vary from a loss of activity with just one freeze–thaw cycle using serum from various fish species [95], compared to conserved bactericidal activity following up to 3 [96] and 5 [97] freeze–thaw cycles with human serum.

Another consideration for the use of complement in neutralisation assays is the choice of cell line. Some cells naturally express complement regulatory proteins which would affect their sensitivity to cytotoxicity. If these cells are used for virus propagation, some viruses can incorporate complement regulatory proteins of the host cell into their lipid membrane during the budding process, which can make them resistant to complement activity [98–100]. Also, a virus may use different cell-entry mechanisms for different cell lines, which could affect the sensitivity to complement-mediated enhancement, as highlighted previously [54]. The complement source is another potential variable. Human, guinea pig, and rabbit sera/plasma are often pooled and used as an exogenous complement source. There are differences between these sources in the potency of complement activity [101, 102]. Some studies use purified complement proteins to recapitulate all, or part, of the complement system. Whilst this approach is considerably more expensive and complex, the concentrations of each protein can be controlled to better understand the mechanisms and underlying dynamics of enhancement. Lastly, whilst the differences between heat-inactivated versus non-heat-inactivated sera/plasma samples can be indicative of complement activity, it is not conclusive. The use of purified complement proteins, or the use of sera/plasma depleted in complement components which can then be reconstituted, as discussed within this review, provide more conclusive evidence of the role of the complement system.

Pseudotype virus neutralisation assays (PNAs) enable higher throughput at lower levels of biocontainment compared to the use of wild-type virus and can maintain strong correlations with gold standard neutralisation assays such as the PRNT, as demonstrated for SARS-CoV-2 [103]. A complement-mediated enhancement of antibody neutralisation titres has been shown for pseudotyped hepatitis C virus (HCV). Meyer et al. [104] demonstrated a ~ 60 to 160-fold increase in human monoclonal antibody neutralisation titres following the addition of guinea pig sera and human sera as sources of complement. This effect was heat labile and dependent on complement component C4, but not factor B. Whilst the addition of complement to PNAs is feasible, there are additional considerations compared to the use of wild-type virus. Firstly, some viral proteins have complement regulatory functions and these may be absent in the pseudotyped virus. Secondly, whilst only mentioned briefly within this review, complement can have intracellular antiviral functions [14] which may lose relevancy with replication-deficient pseudotype virus and the need for the backbone of an alternative virus [105]. A summary of the techniques described within this review to determine the complement-mediated enhancement of antibody neutralisation potency are summarized in Table 2.

**Table 2:** A summary of the primary methods used to determine a complement-mediated enhancement of neutralising antibody potency described in the consulted studies

Summary of consulted studies: methods to study the complement-mediated enhancement of antibody neutralisation potency	
Example	Description
1. Antibody source	
Heat-inactivated immune sera/plasma	Heat-inactivated sera/plasma from convalescent or vaccinated individuals can be used as a source of antibodies to demonstrate complement-mediated enhancement. The samples are heat inactivated to prevent confounding effects of the complement system, which is then replenished using an exogenous complement source
Purified antibodies	Purified IgG and IgM antibodies from sera/plasma can be used as a source of antibodies. Whilst this may be less representative of natural infection compared to the use of sera/plasma, there are no confounding effects of complement or other proteins, the antibody concentrations are easily controlled, and the antibodies of interest are more readily characterized
2. Exogenous complement source	
Human and animal sera/plasma	Guinea pig, rabbit, and human sera/plasma are commonly used as exogenous sources of complement with final concentrations for in vitro studies typically in the range of 5–20%. The exogenous complement source can be heat inactivated as a control measurement to demonstrate that the enhancement is heat labile, which is indicative of complement activity. However, further experiments should ideally be conducted to confirm complement activity i.e., depletion of complement proteins from the sera/ plasma or the use of purified complement proteins
Complement-depleted sera/plasma	Complement proteins can be depleted from the sera/plasma sources. The depleted protein can then be replenished to demonstrate that a loss/gain of neutralisation can be attributed to particular complement proteins. Depleted sera/plasma can also be used to indicate the mechanism of action, i.e., if reconstitution of C3, but not C5, is essential for enhanced neutralisation then the mechanism is unlikely to be MAC mediated
Purified complement proteins	Purified complement proteins can be used at physiological concentrations. This method is considerably more expensive compared to the use of sera/plasma, but clearly demonstrates the role of complement proteins and enables further scrutiny of the mechanism of action and reaction kinetics
3. Technique	
Plaque reduction neutralisation test (PRNT)	PRNTs are considered the gold standard for determining neutralisation titres. The addition of an exogenous complement source to PRNTs has enhanced antibody neutralisation titres against a range of viruses including Rubella virus [37], Hantavirus [45], and EBOV [43]. Cytotoxicity may be more problematic for PRNTs if long incubation times are required to generate plaques
Pseudotype virus neutralisation assays (PNAs)	PNAs can be higher throughput and conducted at lower levels of biocontainment compared to the use of wild-type virus. The addition of an exogenous complement source to PNAs can result in a significant increase to antibody neutralisation titres, as shown for pseudotyped HCV [104]
Electron microscopy	Electron microscopy has been successfully used to show the aggregation [25] and lysis [11] of virus particles in the presence of complement and immune sera/plasma or purified antibodies
Comet tail inhibition assay	The addition of an exogenous complement source to comet tail inhibition assays showed a stronger correlation to in vivo findings for the investigation of therapeutics against VACV [65]
In vivo	The in vivo studies described within this review utilised mouse [23, 25, 46, 65] and NHP [75] models to demonstrate antibody efficacy, and correlations with neutralisation assay results were strengthened with the addition of an exogenous complement source. Animal models could be used utilized further to show the impact of complement in vivo, such as the use of C3 knockout mice or mice/guinea pigs treated with cobra venom factor

Each method includes an “Antibody Source” which is the sample of interest that contains the antibodies being investigated for complement-dependency. Then an “Exogenous Complement Source” is used to replenish (if heat-inactivated) or introduce (if using purified antibodies) the complement system to the “Antibody Source”. Lastly, various “Techniques” can then be used to study the effect of the “Antibody Source” and “Exogenous Complement Source” on virus neutralisation

It is also important to highlight that the role of the complement system in pathogenesis and disease is often complex. Whilst this review puts forward the lesser-known argument regarding enhancement of antibody neutralisation and some of the potential benefits of the complement system, it may also contribute to pathogenesis and the severity of disease [14, 106]. The pro-inflammatory and chemotactic response of complement is often shown to have negative implications for disease severity, but it is also able to regulate many cell-mediated effects and the development of adaptive immunity which are often vital for protection. Complement activation can also occur via the antibody-independent lectin and alternative pathways. Whilst the lectin pathway has not currently been shown to contribute to the enhancement of antibody-dependent neutralisation discussed in this review, it can mediate virus neutralisation, independent of antibodies, through some of the mechanisms described [14]. Lastly, many viruses employ complement regulatory/evasion mechanisms to establish infection resulting in pathogenesis [14, 80]. This adds to the intricacy of the subject but can also provide potential avenues for new therapeutics.

Whilst the focus of this review is on the complement system as a serum component, the complement system can also have physiological relevance in the respiratory tract and, to a lesser extent, the saliva. In a study by Watford et al. [107], components of the classical and alternative complement pathways were present in the lung lavage fluid of healthy volunteers. The classical pathway was shown to be functionally active and capable of inducing lysis at ~ 39% of the magnitude of serum activity, whilst C3 deposition was ~ 16% compared to serum activity. Non-immune cells in the respiratory tract such as fibroblasts, mesothelial cells, goblets cells, mucous cells, club cells, AT2 cells, and alveolar type II epithelial cells can also synthesise complement components and its regulatory proteins in the homeostatic state [108–110]. Immune cells such as monocytes, macrophages, and dendritic cells which can reside in the lung, or infiltrate the respiratory tract during infection, can synthesise all complement proteins and regulators required for functional activity and can interact with complement proteins upon binding to specific receptors [111]. Immune proteins such as salivary scavenger and agglutinin (SALSA) are found in the oral cavity (as well as the lungs and other mucosal surfaces) and can activate the classical and lectin pathways of the complement system [112]. In the healthy state, some complement proteins have been detected in human saliva and shown to be functional (C3, C4, factor B) [113]. Inflammation and mechanical damage can enable the transfer of complement proteins from the blood into the saliva to facilitate full complement activity. IgM and IgG specific to some viruses, such as measles virus [114], SARS-CoV-2 [115], and EBOV [116], are also present at detectable levels in oral fluids. The presence of IgM and IgG in saliva primarily occurs via passive transfer from the blood circulation but can also be locally produced [117]. Of the immunoglobulins, IgA predominates in saliva. However, it has only been shown to activate the lectin and alternative complement pathways in the polymeric form, when using purified/recombinant IgA or IgA from serum, coated on microtiter plates. This could be due to slight denaturation when bound to the plastic, or differences in glycosylation between polymeric and monomeric IgA [118–121]. Despite the implications of complement and virus neutralisation in the respiratory tract, there is a relative paucity of knowledge regarding its impact on viral infection.

In summary, neutralisation assays are critical for investigating antibody efficacy/potency in vitro and potential efficacy in vivo. Limitations to these assays, such as the absence of potential Fc-mediated effector functions, are generally recognized but comparatively under-researched. And of these functions it is a lesser known phenomenon that the complement system can directly enhance neutralisation titres, and that common methods for conventional neutralisation assays abrogate this mechanism. This review provided evidence for this phenomenon, explored our current understanding of the underlying mechanisms, highlighted the current limitations in our understanding, and finally explained how these methods can be applied to benefit future research with applications to vaccine and therapeutic development. This can be of particular importance for: the development of therapeutics where current options are limited and/or threatened by the emergence of novel variants; developing a comprehensive understanding of the relationship between neutralising antibodies and protection; accelerating vaccine licensure through better defined CoPs; and evaluating immune responses following vaccination and/or infection.

## Data Availability

Not applicable.
